# Fostering eco-friendly excellence: exploring the relationship between green human resource practices and organizational environmental performance as perceived by nurses: a cross-sectional study

**DOI:** 10.1186/s12912-025-03097-7

**Published:** 2025-05-07

**Authors:** Amal Diab Ghanem Atalla, Ayman Mohamed El-Ashry, Sabrein Mahmoud Khalifa Khattab

**Affiliations:** 1 Nursing Administration, Faculty of Nursing, Alexandria University, Alexandria, Egypt; 2 Psychiatric and Mental Health Nursing, Faculty of Nursing, Alexandria University, Alexandria, Egypt

**Keywords:** Green, Human resource management practices, Organizational, Environmental performance, Nurses

## Abstract

**Aim:**

This study investigates the association between green human resource practices and organizational environmental performance among nurses at Alexandria Main University Hospital.

**Design:**

A cross-sectional descriptive design following STROBE guidelines examined the relationship between green human resource practices and organizational environmental performance among nurses.

**Methods and tools:**

Staff nurses employed in all inpatient medical, surgical, and critical care units at Alexandria Main University Hospital (*n* = 745) comprised the participants, representing all target demographics. The nurses filled out the Organizational Environmental Performance Scale and the Green Human Resources Practices Questionnaire. Age, gender, education, and nursing experience were among the demographic details gathered. The data collection period was three months, from February 1st, 2023, to May 1st, 2023. Every query from the nurses was addressed, and justifications were provided. The data was analyzed using statistical techniques such as stepwise regression, ANOVA, t-tests, and Pearson correlation.

**Results:**

60.7% of the studied nurses perceived low Green Human Resources Practices (63.27 ± 8.14). Furthermore, the majority of the nurses who participated in the study (90.1%) believe that environmental performance is at a moderate level, with a mean score of 73.86 ± 15.54. According to linear regression, the Green Human Resources Practices of the nurses under study can account for about 17.5% of the explained variance of their perceived environmental performance; this is a significant model (F = 157.939 & *p* < 0.001).

**Conclusion:**

The study highlights the importance of the perceived adoption of Green Human Resources Practices in supporting environmental performance. Addressing demographic factors and fostering a supportive work environment is crucial for optimizing organizational environmental performance.

**Nursing implications:**

Insights from this study can enlighten battered interventions and policy pronouncements to augment nursing practice, organizational growth, and healthcare outcomes in Egypt. Green HR practices have consequences for nursing, such as enabling nurses to spearhead sustainability projects, encouraging environmentally friendly patient care techniques, and raising employee involvement through incentives and training. Nurses can make a substantial contribution to better environmental performance in healthcare settings by incorporating these practices. In the end, this promotes a sustainable culture that is advantageous to patients and the environment.

**Clinical trial number:**

Not applicable.

## Introduction

Protecting and conserving natural resources in the ecosystem are seen as a top priority by decision-makers and managers in various organizations [1]. In the healthcare sector, there is increased competition, leading managers to seek new ways to optimize their essential resources, particularly human resources (HR) [2]. HR is crucial for implementing policies and practices that stimulate sustainable performance and competitive advantage [3]. Organizations are increasingly adopting environmentally friendly activities to balance resource consumption and economic development, which can lead to operational improvement, economic gain, and competitive advantage [4].

Healthcare organizations are concentrating on environmental sustainability, and new approaches to human resource practices have been developed to support green and sustainable initiatives that turn out to be the agenda of decision-makers in the 21st century [5]. The creation of innovative human resource management (HRM) techniques that support green initiatives, which have become a priority for 21st-century decision-makers, is a result of healthcare businesses’ growing emphasis on environmental sustainability. By encouraging eco-friendly behaviors, creating policies that promote recycling and waste reduction, and raising awareness and training among healthcare personnel, effective green human resource practices can make a substantial contribution to waste management. This connection is critical because the successful implementation of sustainable waste management solutions depends on a staff that is properly trained. However, political reforms that could upset stability, undeveloped financial markets that restrict investment in sustainability projects, and trade restrictions that restrict access to green resources and technologies make it particularly difficult for developing nations to adopt these green practices. To promote environmental sustainability, these countries must overcome the obstacles that make it difficult to implement efficient waste management and other sustainable practices in the healthcare industry [6–8].

Healthcare organizations are facing increasing pressure to embrace eco-friendly practices, but there is a need for better research on green human resource practices in developing nations. Growing public awareness of environmental issues, regulatory requirements, and stakeholder expectations, including those of patients, governments, and advocacy groups that place a high priority on sustainability in healthcare delivery, are all putting pressure on the healthcare industry to adopt eco-friendly practices. Global movements for sustainable development and climate action, which demand that businesses lessen their environmental impact, are increasing this pressure. Since the existing literature may not sufficiently address the particular socio-economic and cultural factors at play, further study on green human resource practices in developing countries is desperately needed to comprehend the special opportunities and problems these environments bring. Research can have significant consequences for better environmental performance in various areas by shedding light on best practices and guiding the creation of customized green human resource plans that successfully incorporate sustainability into healthcare operations [9]. Human resource managers are now integrating environmental awareness requirements into job descriptions and interview processes to attract candidates who share the organization’s environmental goals. Among healthcare professionals, nurses are especially well-suited to play a role in reducing waste and controlling emissions [10].

Green human resource practices offer a tangible approach for organizations to cultivate a workforce that can enhance the organization’s environmental performance (EP) and contribute to sustainable development [11–13]. These practices entail activities that yield favorable environmental results [14]. Once recruited and proficient, employees are kept motivated through performance evaluation and compensation systems that provide avenues for expanding environmental performance [10, 15]. GHRM practices center on nurturing the skills of environmentally conscious employees, encouraging them to strive for improvements in environmental performance, and offering opportunities for green initiatives [10].

These practices integrate environmental thinking into HR activities such as recruitment, selection, training, and leadership development. Motivation is maintained through performance measurement and reward systems that emphasize opportunities for environmental performance improvement [16]. GHRM practices focus on reducing waste and improving organizational efficiency, thus enhancing EP [17, 18].

EP is clearly defined as an organization’s dedication and commitment to safeguarding the environment, which is demonstrated through measurable operational parameters that align with predetermined environmental precautionary limits [19]. A comprehensive assessment of EP encompasses aspects such as reducing incidents, ongoing enhancements, recycling efficiency, how stakeholders perceive the organization, conducting audits, minimizing waste, and realizing cost savings [20]. Human resource managers have a vital role to play in attaining these EP objectives by recruiting, training, evaluating, and incentivizing an environmentally conscious workforce [10].

Numerous scholars have researched the connections between green human resource practices and an organization’s EP [5, 10]. Green human resource practices have a positive impact on EP by reducing waste and enhancing organizational efficiency [21]. These practices can also encourage employees to engage in environmentally friendly behavior, voluntarily contributing to improved organizational performance [22, 23]. However, it’s worth noting that while the link between green human resource practices and EP is well-established, any study of how environmentally conscious employees implement these green HRM initiatives without considering their effect on EP remains incomplete. Recent studies have emphasized the necessity for further research into the relationship between an organization’s green human resource practices and its EP [24–26].

The study’s contribution is its particular focus on the healthcare context, especially through the perspectives of nurses, even though it may draw from previous research on the connection between environmental performance and green human resource practices from other sectors. Because healthcare has particular operational challenges and regulatory frameworks that affect the application of green human resource principles, this method fills a large vacuum in the literature. The study’s analysis of nurses’ perspectives offers a sophisticated grasp of how these procedures are viewed and carried out in a clinical context, which can guide focused tactics for improving sustainability in healthcare. Furthermore, this study can assist healthcare institutions in creating customized green human resource programs that better suit the requirements and experience of nursing staff, ultimately resulting in better patient care results and environmental performance in poor countries.

### Theoretical background

When examining the relationship between green human resource practices and organizational environmental performance, there isn’t a single overarching theory that universally explains the results. However, several theoretical perspectives and frameworks can provide a foundation for understanding this relationship. The Resource-Based View (RBV) theory suggests that unique resources and capabilities within an organization contribute to its competitive edge. In the realm of green human resource practices and environmental performance, this perspective implies that effective strategies, such as eco-friendly training initiatives, employee participation in green projects, and policies with an environmental focus, can act as significant resources that boost the environmental performance of a company [27–29]. The study is informed by the theoretical framework of legitimacy theory, which emphasizes how organizations implement green human resource practices to conform to social norms and stakeholder expectations. Healthcare organizations can increase their credibility and trust with patients, providers, and the community at large by putting environmental measures into practice. The significance of perceived legitimacy in promoting organizational commitment to sustainability is emphasized by this approach. In the end, it presents green human resource practices as a tactical method that helps healthcare businesses deal with stakeholder demands and succeed in the long run [28, 29].

### Significance of the study

Since nurses play a significant role in implementing sustainability initiatives in healthcare settings, as focused in Egypt Vision 2030, evaluating green human resource practices from their points of view is imperative. Their observations, which show how these methods are viewed and used in clinical settings, offer insightful information on their efficacy and applicability. This method captures the diverse perspectives of nurses while acknowledging the subjective aspect of human resource practices. Furthermore, it fills in a significant gap in the literature because studies frequently ignore the particular setting of the healthcare industry. Organizations can create focused policies and training initiatives that improve participation in sustainability initiatives by knowing nurses’ perspectives. The ultimate goal of this research is to enhance environmental performance in healthcare and promote change that can be put into practice.

Although Green Human Resource Practices (GHRP) in healthcare settings have been the subject of earlier research, my study focuses on nurses in Egypt, a context that has not gotten much attention in the literature on sustainability and HRM. This study offers fresh perspectives on how nurses view and contribute to organizational environmental performance, which is important given the particular environmental issues and organizational dynamics in Egyptian healthcare. To distinguish my work from previous research and highlight its value in comprehending GHRP in the context of Egyptian nursing, I will further polish this portion.

Because this study emphasizes the vital role that Egyptian nurses play in putting green human resource practices into practice in healthcare settings, this study is imperative to them. Nurses can improve their professional skills and support a sustainable culture in a fast-evolving healthcare system by learning about and perceiving eco-friendly activities. The results may also have an impact on healthcare policies and procedures, encouraging improved environmental stewardship and resource management. In addition to improving patient outcomes and overall care quality, this alignment with global sustainability goals can increase nurses’ pride and involvement. In the end, the sequence equips Egyptian nurses to lead their profession in environmental activism.

### Aims of the study

- Examine the relationship between Green Human Resource Practice and Organizational Environmental Performance as perceived by nurses.
- Examine the differences in perceptions of Green Human Resource Practices and Organizational Environmental Performance across demographic groups among nurses.

### Research question

- What is the relationship Between Green Human Resource Practices and Organizational Environmental Performance as Perceived by nurses?
- Do perceptions of Green Human Resource Practices and Organizational Environmental Performance vary significantly across different demographic groups (age, gender, years of experience, qualification) among nurses?”

## Materials

### Design and setting

The research employed a descriptive correlational research design. The study took place in all inpatient medical, surgical, and critical care units within the Alexandria Main University Hospital. This university hospital is equipped with a total of 6,825 beds. Specifically, the surgical care units, each specializing in different areas, account for a total of 773 beds. The medical care units, also with various specialties, encompass 952 beds. Additionally, there are 100 beds allocated to critical care units. Within the surgical care units, there are 17 different specialties represented. The medical care units consist of 25 specialties, and the critical care units have 13 specialties. It has the utmost sum of healthcare suppliers. It has diverse categories and qualifications of nurses, for instance, professional, technical, and diploma nurses. It accepts patients from all governorates in Egypt. It delivers wide-ranging healthcare services as an inpatient, outpatient, critical, intensive, and emergency care, radiological, laboratory, and physical therapy service area. Data was collected over three months, from the beginning of April to the end of July 2023.

### Participants

This is a convenient sampling method where we choose to look at the entire population (i.e., the total population) that fits a certain set of conditions (inclusion criteria). All target populations of nurses were included in the study. The participants were chosen to gather the required data from among the 745 nurses who worked in the aforementioned unit. They were more familiar with the hospital system and administrative rules, policies, and regulations because they had been employed in the aforementioned units for at least a year. They were also willing to engage in the study and were there when the data was being collected.

### Ethical considerations

Before the study, approval for conducting the study was obtained from the Research Ethics Committee (REC) at the Faculty of Nursing, Alexandria University. The participants were provided with detailed information about the study’s purpose, and their informed consent was obtained. To ensure confidentiality and anonymity, each questionnaire was assigned a unique code number instead of using personal identifiers. The nurses were assured that their data would be kept confidential and used solely for research. Furthermore, the participants were informed of their right to withdraw from the study without facing any negative consequences.

## Study measurement tools

### Two tools were used in the current study as follows

#### Tool I: Green Human Resources Practices Questionnaire (GHRQ)

This survey tool was designed by Abou-AL-Ross & Abu Mahadi (2017) [29]. It consists of 51 items divided into the following six dimensions: Green recruitment and selection (7 items), green training and development (10 items), green performance appraisal (8 items), reward and compensation (7 items), participation and empowerment (8 items) and green organizational culture (11 items). Participants responded to items on a 10-point Likert scale ranging from 1 strongly disagree to 10 strongly agree. Scoring for the total scale and subscales ranged from 51 to 510. Scores ranged from 51-< 204, reflecting a low perception of GHRM practices. Scores ranged from 204-<357, reflecting a moderate perception of GHRM practices, and scores ranged from 357 to 510, reflecting a high perception of GHRM practices. The English version of the GHRQ was translated into Arabic by two bilingual translators to make it suitable for Arabic-speaking audiences. This Arabic version was then independently retranslated into English by another pair of translators to verify its accuracy. A Confirmatory Factor Analysis (CFA) was conducted on the translated version. The model’s goodness of fit indices, such as 0.821, TLI of 0.861, and RMSEA of 0.100, suggest that the model is a good fit.

#### Tool II: Organizational environmental performance scale

It was developed by Abou-AL-Ross & Abu Mahadi (2017) [29]. It consisted of 15 items to assess Organizational Environmental Performance as perceived by the studied nurses. The responses were rated on a 10-point Likert scale ranging from 1 strongly disagree to 10 strongly agree. The overall score ranges from 15 to 150. A score ranging from 15 to 59 reflects that nurses perceived a low level of Organizational Environmental Performance, from 60 to 104 reflects a moderate level of Organizational Environmental Performance, and a score ranging from 105 to 150 reflects a high level of career motivation as perceived by nurses. The English version of the Organizational Environmental Performance Scale was translated into Arabic by two bilingual translators to make it suitable for Arabic-speaking audiences. This Arabic version was then independently retranslated into English by another pair of translators to verify its accuracy. A Confirmatory Factor Analysis (CFA) was conducted on the translated version. The model’s goodness of fit indices, such as 0.810, TLI of 0.891, and RMSEA of 0.100.

In addition, the nurses’ demographic data sheet was developed by the researchers to collect data about their age, gender, current working unit, and years of experience.

### Face validity and reliability

The two tools used in the study were modified and translated into Arabic and then translated back into English to ensure accuracy. These tools were then reviewed and tested for content validity by a panel of three experts, including two professors from the Faculty of Nursing at Alexandria University and one professor from the Faculty of Nursing at Damanhour University. The experts provided feedback on the content, question nature, and clarity of the items, which was taken into consideration to ensure the accuracy and validity of the study. The reliability of the tools was also assessed by measuring the internal consistency of the items using Cronbach’s alpha coefficient test. The results showed that both tools were reliable, with a Cronbach’s alpha coefficient of 0.90 for the Green Human Resources Practices Questionnaire and 0.88 for the Organizational Environmental Performance Scale.

### Pilot study

A pilot study was conducted with a subset of staff nurses, specifically 10% of them (*n* = 75), within the same setting mentioned earlier. The purpose was to confirm the clarity and practicability of the survey items, pinpoint any potential obstacles or challenges that might arise during data collection, and determine the amount of time required to complete the assessment tools. Nothing had to be changed. It’s important to note that individuals who took part in the pilot study were not included in the main study sample.

### Data collection

The researchers obtained official approval from the Dean of the Faculty of Nursing and the board at the study Hospital before distributing the questionnaire to participating nurses. The data collection process took place with the permission of the nurse managers of the respective units during agreed-upon break times. All participants were informed about the study’s objective, and each nurse was given detailed instructions before spending approximately 15–20 min to finalize the questionnaire. All questions were answered, and explanations were given accordingly. The data collection period was three months, from February 1st, 2023, to May 1st, 2023. Every query from the nurses was addressed, and justifications were provided.

### Data analysis

The collected data were analyzed using SPSS version 20. Descriptive statistics such as frequencies, means, standard deviations, and percentages were employed to quantify the demographic and work-related characteristics of the participants. Inferential statistics, including the Student’s t-test and analysis of variance (ANOVA), were utilized to compare the means of GHRM Practices and EP between different groups based on socio-demographic characteristics. The correlation coefficient was also calculated to examine the relationship between GHRM Practices and EP. Also, regression analysis (R2) was run to assess if the independent variable (the GHRM Practices) might predict the dependent variables (EP). With an alpha of 0.05, all statistical analyses were conducted.

## Results

Table 1 shows that 42% of the studied nurses were less than 30, and 82.6% were male. More than 42% of them were working in surgical care units. 38.7% of the studied nurses hold nursing school diplomas. Also, 29.1% of them had 11 to less than 20 years of experience in the nursing profession.

**Table 1:** Distribution of the studied nurses according to demographic data (*n* = 745)

	No.	%
**Age (years)**		
< 30	313	42.0
30–40	219	29.4
41–50	156	20.9
> 50 y	57	7.7
Mean ± SD	35.39 ± 9.46	
**Sex**		
Male	615	82.6
Female	130	17.4
**Unit**		
Surgical	316	42.4
Medical	227	30.5
ICU	202	27.1
**Qualification**		
Nursing school diploma	288	38.7
Technical nursing institute	191	25.6
Bachelor of nursing science	266	35.7
**Years’ experience of nursing**		
< 5	112	15.0
5–10	205	27.5
11–20	217	29.1
> 20	211	28.3
Mean ± SD	14.24 ± 8.73	
**Years’ experience of unit**		
< 5	446	59.9
5–10	188	25.2
11–20	37	5.0
> 20	74	9.9
Mean ± SD	6.61 ± 7.14	
**Shift**		
Fixed morning	211	28.3
Rotating morning and evening	184	24.7
Rotating morning and evening and night	188	25.2
Fixed night	162	21.7

SD: Standard deviation

As illustrated in Tables 2 and 60.7% of the studied nurses perceived low Green Human Resources Practices (63.27 ± 8.14). Moreover, the highest mean percent score of the GHRM practices dimension is listed for training and development (70.62 ± 6.66), followed by performance management and appraisal (67.11 ± 9.31). On the other hand, the lowest mean percent score was recorded for participation and empowerment (51.65 ± 14.39), followed by recruitment and selection (58.14 ± 10.07). Moreover, the majority of the studied nurses (90.1%) perceive a moderate level of Environmental performance with a mean score equal to 73.86 ± 15.54.

**Table 2:** Distribution of the studied nurses according to their levels and mean percent score of green human resources practices’ questionnaire and organizational environmental performance scale (*n* = 745)

	Low (< 33.3%)		Moderate (33.3 – <66.6%)		High (≥ 66.6%)		Total score
	No.	%	No.	%	No.	%	Mean ± SD
Recruitment and selection	10	1.3	586	78.7	149	20.0	23.28 ± 2.82
Training and development	4	0.5	228	30.6	513	68.9	38.25 ± 2.66
Performance management and appraisal	5	0.7	449	60.3	291	39.1	29.47 ± 2.98
Reward and compensation	76	10.2	303	40.7	366	49.1	24.72 ± 4.15
Participation and empowerment	149	20.0	522	70.1	74	9.9	24.53 ± 4.61
Organizational culture	9	1.2	364	48.9	372	49.9	39.83 ± 9.28
Total green human resources practices’ questionnaire	4	0.5	452	60.7	289	38.8	180.08 ± 16.61
Organizational environmental performance scale	8	1.1	66	8.9	671	90.1	59.32 ± 9.32

SD: Standard deviation

Table 3 exhibits a highly statistically Significant positive correlation between the overall GHRM practices and the overall environmental performance (*r* = 0.419, P = < 0.001).

**Table 3:** Correlation between green human resources practices’ questionnaire and organizational environmental performance scale (*n* = 745)

		Tool I							Organizational environmental performance
		Recruitment and selection	Training and development	Performance management and appraisal	Reward and compensation	Participation and empowerment	Organizational culture	Total GHRM	
Recruitment and selection	r		0.125^*^	0.112^*^	0.346^*^	0.893^*^	0.184^*^	0.647^*^	0.212^*^
	p		0.001^*^	0.002^*^	< 0.001^*^	< 0.001^*^	< 0.001^*^	< 0.001^*^	< 0.001^*^
Training and development	r			0.292^*^	0.471^*^	0.152^*^	0.258^*^	0.538^*^	0.323^*^
	p			< 0.001^*^	< 0.001^*^	< 0.001^*^	< 0.001^*^	< 0.001^*^	< 0.001^*^
Performance management and appraisal	r				0.238^*^	0.148^*^	0.083^*^	0.392^*^	0.174^*^
	p				< 0.001^*^	< 0.001^*^	0.023^*^	< 0.001^*^	< 0.001^*^
Reward and compensation	r					0.503^*^	0.115^*^	0.630^*^	0.126^*^
	p					< 0.001^*^	0.002^*^	< 0.001^*^	0.001^*^
Participation and empowerment	r						0.100^*^	0.661^*^	0.219^*^
	p						0.006^*^	< 0.001^*^	< 0.001^*^
Organizational culture	r							0.703^*^	0.371^*^
	p							< 0.001^*^	< 0.001^*^
Green human resources practices’ questionnaire	r								0.419^*^
	p								< 0.001^*^
Organizational environmental performance scale	r								
	p								

r: Pearson coefficient *: Statistically significant at *p* ≤ 0.05. (GHRM) Green Human Resources Practices’ Questionnaire

Table 4 displays a statistically significant difference between overall green HRM practices and studies nurses’ age (F = 7.241, P = < 0.001); the highest mean score percent (67.28 ± 7.77) was related to nurses in the age group above 50 years old, while the lowest mean score percent (62.09 ± 7.88) was related to the nurses under 30 years old.

**Table 4:** Relation between green human resources practices’ questionnaire and organizational environmental performance scale with demographic data (*n* = 745)

Demographic data	Green human resources practices’ questionnaire	Organizational environmental performance scale
	Mean ± SD	Mean ± SD
**Age (years)**		
< 30	62.09 ± 7.88	73.04 ± 14.59
30–40	63.63 ± 8.42	74.54 ± 15.16
41–50	63.68 ± 7.91	73.48 ± 17.68
> 50 y	67.28 ± 7.77	76.78 ± 76.78
**F(p)**	**7.241** ^*^ **(< 0.001** ^*^ **)**	**1.136 (0.334)**
**Sex**		
Male	63.32 ± 8.27	74.15 ± 15.44
Female	63.04 ± 7.53	72.47 ± 15.99
**t(p)**	**0.363 (0.717)**	**1.120 (0.263)**
**Unit**		
Surgical	62.94 ± 9.02	73.97 ± 16.12
Medical	63.09 ± 6.92	72.92 ± 14.08
ICU	64.0 ± 7.95	74.75 ± 16.16
**F(p)**	**1.122 (0.326)**	**0.754 (0.471)**
**Qualification**		
Nursing school diploma	62.74 ± 8.19	72.89 ± 15.81
Technical nursing institute	62.98 ± 8.31	73.12 ± 16.01
Bachelor of nursing science	64.07 ± 7.94	75.44 ± 14.81
**F(p)**	**2.018 (0.134)**	**2.168 (0.115)**
**Years’ experience**		
< 5	61.93 ± 7.70	70.80 ± 19.10
5–10	62.03 ± 8.41	74.09 ± 11.52
11–20	63.78 ± 8.02	74.53 ± 15.13
> 20	64.68 ± 8.0	74.57 ± 17.08
**F(p)**	**5.053** ^*^ **(0.002** ^*^ **)**	**1.749 (0.156)**
**Years’ experience of unit**		
< 5	62.38 ± 8.10	73.04 ± 15.12
5–10	63.72 ± 8.03	73.48 ± 17.19
11–20	65.37 ± 7.99	79.05 ± 12.51
> 20	66.51 ± 7.82	77.21 ± 77.21
**F(p)**	**6.878** ^*^ **(< 0.001** ^*^ **)**	**3.002** ^*^ **(0.030** ^*^ **)**
**Shift**		
Fixed morning	63.41 ± 8.87	73.87 ± 15.54
Rotating morning and evening	63.16 ± 8.22	72.63 ± 17.18
Rotating morning and evening and night	64.54 ± 7.16	75.37 ± 14.15
Fixed night	61.77 ± 7.96	73.50 ± 15.09
**F(p)**	**3.404** ^*^ **(0.017** ^*^ **)**	**1.010 (0.388)**

SD: Standard deviation t: Student t-test F: F for One way ANOVA test

p: p value for comparison between the studied categories. *: Statistically significant at *p* ≤ 0.05

The result shows a significant difference between green HRM practices and years of experience of the nurses (*P* = 0.002). The nurses with more than 20 years of experience in the nursing profession had the highest mean score (64.68 ± 8.0), while the nurses with less than 5 years of experience had the lowest mean score (61.93 ± 7.70). A significant difference was exhibited between overall green HRM practices and studies of nurses’ experience in the current working unit (*P* < 0.001). The nurses with more than 20 years of experience in the current working unit had the highest mean score (66.51 ± 7.82), whereas the nurses with less than 5 years of experience had the lowest mean score (62.38 ± 8.10). Only a significant difference existed between the studied nurses’ EP and their years of experience in the current working unit (*P* = 0.030).

Table 5 indicates univariate analysis linear regression coefficient value between the GHRM practices as the independent variable and environmental performance as a dependent variable. The value of the regression coefficient was (R2 = 0.175). This means that Green Human Resource Management (GHRM) practices account for approximately 17.5% of the variation in how nurses perceive environmental performance within their organization where the model is significant (F = 157.939 & *p* < 0.001).

**Table 5:** Linear regression for the GHRM practices as independent variable and organization environmental performance as a dependent variable

	B	Beta	t	*p*	95% CI	
					LL	UL
Green human resources practices’ questionnaire	0.799	0.419	12.567^*^	< 0.001^*^	0.674	0.924
R^2^ = 0.175, F = 157.939^*^,*p* < 0.001^*^						

F, p: f and p values for the model; R^2^: Coefficient of determination; B: Unstandardized Coefficients; Beta: Standardized Coefficients; t: t-test of significance; CI: Confidence interval; LL: Lower limit; UL: Upper Limit; *: Statistically significant at *p* ≤ 0.05

## Discussion

Determining the relationship between GHRM practices and environmental performance in healthcare organizations is crucial for providing valuable insights for strategic HR planning; this helps healthcare organizations align their human resource practices with organizational goals and objectives to maximize staff performance. In this vein, Abou Hashish& Ghanem Atalla (2023) recommended that hospital and nurse managers build and maintain a supportive work environment, thus enhancing organizational environmental performance [30].

The current study results revealed that nurses needed a higher level of perception regarding green human resource practices. This could be due to nurses’ frontline experience, which enables them to see firsthand how environmental measures affect patient care and safety, and nurses may have a positive opinion of green human resource practices. Nowadays, a lot of nursing programs include sustainability in their curricula, giving nurses the skills they need to adopt environmentally friendly procedures. The significance of a healthy environment for the best possible patient outcomes is further reinforced by their strong ethical commitment to holistic patient care. Additionally, nurses naturally broaden their advocacy to include sustainability measures because they are champions for patient and community health. Finally, nurses’ perceptions of sustainability’s importance are improved when healthcare companies prioritize it, which encourages them to actively participate in these practices.

These results are inconsistent with Hammad (2015), who found that green human resource practices. are low and should be enriched to achieve sustainable aims and attain good environmental performance [31]. This result also contradicts Mamdouh & Samir (2022), who found a moderate perception of nurses toward green HRM practices [32].

Concerning Environmental performance, the nurses perceived a moderate level of environmental performance. This could be due to several factors, including a lack of competent knowledge about the entire extent of sustainability measures within their workplaces, which may contribute to nurses’ perceptions of a modest level of environmental performance. They might be aware of certain environmentally friendly actions, but a lack of education and training may cause them to not fully comprehend how these efforts affect environmental performance as a whole. Their capacity to engage in and evaluate environmental projects can also be hampered by structural obstacles, including a lack of organizational support, a lack of resources, or a failure to adequately incorporate sustainability into daily operations. Furthermore, a moderate opinion may be a reflection of the difficulties nurses encounter in balancing their clinical duties with sustainability objectives, resulting in a cautious or unsure assessment of the environmental impact of their business.

This is confirmed by Hilton et al. (2023) & Dubey, Pathak, & Sahu (2023), who concluded that if nurses perceive their leaders as competent, supportive, and capable of making sound decisions, it can contribute to a moderate to high perception of organizational performance [33, 34]. This result is inconsistent with Farghaly & Abou Zeid (2023), who found that nurses perceived high organizational performance [35].

The current study results revealed a strong positive relationship between GHRM practices and environmental performance; this result is consistent with prior research by Adeel et al. (2022), who found a significant positive relationship between green HRM practices and EP [36]. This could be because the effective dissemination of environmental knowledge and values through green training and development boosts the EP based on nurses’ skills and motivation, thus constructing a prospect for nurses to engage in environmental system development and augmenting the environmental infrastructure [37]. Ziyadeh et al. (2023) found that hospitals that implemented GHRM practices, such as staff immersion in environmental sustainability initiatives, training and development, and performance management, had higher levels of environmental sustainability [38].

Moreover, this research showed a strong positive correlation between all dimensions of GHRM and environmental performance. These results agreed with Masri & Jaaron (2017 and Kuo et al. (2022), who found that the correlation coefficient of the relationship between Green Recruitment and Selection and EP [39, 40].

Concerning the sociodemographic characteristics of nurses and green HRM practices, the current study results revealed a significant difference between overall green HRM practices and studies of nurses’ age, total years of experience in the profession, and experience in the work unit. The older nurses with more than 20 years of experience had the highest mean score percentage, while the younger nurses with less than 5 years of experience had the lowest mean score percentage. This could be because older nurses had more exposure to training and development in environmental issues and sustainability initiatives, leading to a change in values, beliefs, and attitudes towards environmental performance and sustainability, greater familiarity, and comfort with green HRM practices. They may become more engaged in environmental performance responsibility compared to younger nurses. Older nurses may be more receptive to these practices and more likely to support and participate in them than younger nurses.

These results are consistent with a study by Chen et al. (2020), who found that nurses with more years of experience had higher levels of environmental knowledge and were more likely to engage in environmentally friendly behaviors [41]. A study by Jeong et al. (2019) investigated the relationship between nurses’ environmental literacy and green nursing behaviors and found that nurses with more years of experience had higher levels of environmental literacy and were more likely to engage in green nursing behaviors [42]. A study by MERCAN & MERCAN 2020 to investigate the relationship between ‘environmental attitudes and behaviors’ and ‘environmental sensitivity and perceived environmental risks’ found that nurses with more experience had more positive environmental attitudes and were more likely to perform environmentally friendly behaviors [43].

### Strengths and limitations

This study has several strengths. Using a cross-sectional design allowed for measuring various variables in the population sample at a specific time, providing accurate data less prone to biases than case series or case reports. The study contributes to understanding GHRM practices and organizational environmental performance as nurses perceive, addressing a topic that has yet to receive much research attention in the healthcare context. However, there are certain limitations to consider. Firstly, the generalization of the findings needs to be improved due to the study being conducted in a single center. Secondly, the study only examined the relationship between nurses’ perceptions of organizational environmental performance as a dependent variable and their perception of GHRM practices, while other variables that may impact organizational environmental performance were not measured. Additionally, using a paper-based questionnaire required significant data entry and cleaning effort. Moreover, the survey’s data, collected at a single time point, could introduce Common Method Variance (CMV), a bias that may overstate variable correlations. The article doesn’t address this limitation, which could compromise the survey’s reliability and validity. Future research should employ multiple data collection points or methods to reduce this risk.

## Conclusion

The studied nurses perceived a low level of GHRM practices and a moderate level of organizational environmental performance. In addition, the study found a strong positive relationship between GHRM practices and environmental performance.

### Implications for nursing management

This study has the potential to enhance the understanding and actions of nursing leaders to pave the way to adopt GHRM practices and subsequently improve organizational environmental performance. The findings of this quantitative study also suggest the need for future qualitative research to gain a deeper understanding of the topic, mainly due to the limited predictability of the outcome variables. Also, as organizational Learning is an active process needed for improving organizational performance (Atalla et al. 2022), it is recommended that simulation training programs and workshops be performed to increase staff awareness regarding GHRM practices [44]. Replicating this study in a broader context that includes multiple hospitals and different levels of care would provide comparative results and facilitate the generalization of the findings. Atalla et al. (2023) advised nursing leaders to adjust calendars to attend nurses’ meetings and listen to their opinions, ideas, views, and concerns, thus enhancing organizational environmental performance [45]. The human-centeredness and caring amenities of the nursing profession make it stand out. Only nurses adhere to particular principles when interacting with patients and their families [46]. The most important indicator of a healthcare organization’s competitive advantage and environmental performance is the staff’s environmental behavior [47]. Additionally, future research endeavors should consider including all healthcare providers to ensure the generalizability of the study findings.

## Data Availability

Data will be available upon reasonable request from the corresponding author.
